# Pressure Ulcer Diagnosis Is Associated with Increased Mortality in Patients with End-Stage Renal Disease: A Retrospective Study

**DOI:** 10.3390/life13081713

**Published:** 2023-08-09

**Authors:** Gabriela A. Duchesne, Jennifer L. Waller, Stephanie L. Baer, Lufei Young, Wendy B. Bollag

**Affiliations:** 1 Department of Medicine, Medical College of Georgia at Augusta University, Augusta, GA 30912, USA; gduchesne@augusta.edu (G.A.D.); sbaer@augusta.edu (S.L.B.); 2 Department of Population Health Sciences, Medical College of Georgia at Augusta University, Augusta, GA 30912, USA; jwaller@augusta.edu; 3 Charlie Norwood VA Medical Center, Augusta, GA 30904, USA; 4 Department of Physiological and Technological Nursing, Augusta University, Augusta, GA 30912, USA; 5 Department of Physiology, Medical College of Georgia at Augusta University, Augusta, GA 30912, USA

**Keywords:** pressure ulcer, dialysis, mortality, chronic wounds, end-stage renal disease

## Abstract

Pressure ulcers are associated with multiple comorbidities and annually affect approximately 3 million Americans, directly accounting for approximately 60,000 deaths per year. Because patients with end-stage renal disease (ESRD) are known to present with unique factors which impair wound healing, pressure ulcers diagnosed in ESRD patients might independently increase the risk of mortality. To investigate the association between pressure ulcer diagnosis and mortality risk in the ESRD population, a retrospective analysis of the United States Renal Data System (USRDS) database was performed. The records of 1,526,366 dialysis patients who began therapy between 1 January 2005 and 31 December 2018 were included. Our analysis showed that the diagnosis of pressure ulcers in this population was independently associated with mortality even after controlling for confounding factors (*p* < 0.001). A Kaplan–Meier survival analysis demonstrated reduced survival in patients with a pressure ulcer diagnosis compared to those without a pressure ulcer diagnosis. These results establish pressure ulcers as a significant independent risk factor for mortality, as well as suggesting several comorbidities as potential risk factors for pressure ulcers in the ESRD population.

## 1. Introduction

Pressure ulcers, which affect over 3 million individuals in the United States annually, are defined as localized skin/soft tissue injuries formed due to prolonged pressure or shear, which impairs local blood flow and results in tissue ischemia [1]. Pressure ulcers are characterized as non-healing or chronic wounds, which do not undergo an orderly healing process due to factors such as inadequate angiogenesis, the ineffective anti-bacterial action of neutrophils, limited re-epithelization and chronic inflammation, as well as an increased likelihood of infection associated with the bacterial colonization of the wound [2]. In 2016, hospital-acquired pressure ulcers alone accounted for at least USD 26.8 billion of healthcare expenditures in the US, and every year, pressure ulcers are estimated to directly account for approximately 60,000 deaths [1,3]. Pressure ulcers also significantly impair the quality of life, and patients hospitalized with pressure ulcers are hospitalized for an average of 11.1 days compared to 3 days in patients without pressure ulcers [4]. Pressure ulcer incidence is associated with older age, as well as comorbidities, including diabetes mellitus, cardiovascular disease, peripheral vascular disease (PVD), and spinal cord injuries [5].

Prior research has revealed that patients with chronic kidney disease (CKD) have increased rates of wound disruption, defined as the reopening of a wound, which is an indicator of poor wound healing, after bariatric surgery compared to populations with normal glomerular filtration rates [6]. Many of the associated risk factors for impaired wound healing, such as diabetes, cardiovascular disease, venous insufficiency, and age, are also risk factors for CKD [7]. Mouse models suggest that the dysfunctional wound healing observed in CKD might be mediated by decreased epithelization and granulation tissue deposition and increased inflammation, as well as decreased cellular proliferation and angiogenesis [8]. Other risk factors unique to end-stage renal disease (ESRD), defined as the requirement for dialysis (hemodialysis or peritoneal dialysis), may include uremia, which occurs in ESRD when blood urea accumulates due to the impairment of the kidney’s filtration activity. Uremia negatively affects fibroblast proliferation, collagen production, and immune system function, all of which may contribute to poor wound healing [7,9].

Patients with ESRD require dialysis for survival and have a higher risk of infection due to multiple factors, including a combination of immune system dysfunctions from uremia, as well as complications associated with their required vascular access [10,11]. In the ESRD population, infectious disease is considered the second leading cause of mortality, only behind cardiovascular disease [12]. Bacterial infections also represent the most common complication of pressure ulcers and, in these populations, can progress to serious infections such as bacteremia, osteomyelitis, and septicemia [13,14]. Pressure ulcer-related bacteremia alone is associated with a mortality rate of approximately 50% [15]. Considering these findings, we hypothesized that the diagnosis of pressure ulcers in patients with ESRD may be independently associated with increased mortality even after controlling for demographics and other comorbidities. In order to address this question, we used the United States Renal Data System (USRDS) database to investigate the association of the diagnosis of a pressure wound in patients on dialysis with the risk of their mortality [16].

## 2. Materials and Methods

### 2.1. Study Cohort

The study population comprised all ESRD patients in the USRDS between the ages of 18 and 100 years at the time of the start of dialysis who began therapy between 1 January 2005 and 31 December 2018. Data for individual (coded) patients were included in this USRDS database. Subjects with missing age, race, sex, ethnicity, missing or unknown incident dialysis modality type, or an unknown access type for those with hemodialysis as the first modality were excluded from the study cohort.

### 2.2. Primary Independent Variable

Our main risk factor of interest was any diagnosis of a pressure ulcer in an individual, defined as having at least one diagnosis of a pressure ulcer captured by Medicare claims by either the hospital, physician/supplier (both inpatient and outpatient) or detailed claims data files using the ICD-9-CM and ICD-10-CM codes in Table S1 of the Supplementary Materials.

### 2.3. Outcome Variable

The primary outcome of interest in this study was the time to death of subjects who died or the time period of observation for those who survived in years. Time to death was defined for all subjects as the number of years from the start of dialysis until death; the time period of observation was the number of years from dialysis initiation to 31 December 2019 (to allow for a follow-up of at least one year). Those who did not die were considered censored observations.

### 2.4. Other Independent Variables

Demographic variables included age at the start of dialysis, race, sex, and ethnicity. Incident dialysis type and access type were determined by the Centers for Medicare & Medicaid Services (CMS) from the Medical Evidence Form 2728. The diagnosis of any pressure ulcer and other comorbid conditions were identified using ICD-9-CM and ICD-10-CM codes from hospital claims in the USRDS dataset. Comorbid conditions that were controlled for included alcohol abuse/dependence (alcohol use), nicotine dependence (tobacco use), spinal injury, malnutrition, and all individual components comprising the Charlson comorbidity index as validated in [17,18] and listed in Supplementary Material Tables S2 and S3.

### 2.5. Statistical Analysis

All statistical analyses were performed using SAS 9.4, and statistical significance was assessed using a significance level of 0.05. Descriptive statistics were determined overall by pressure ulcer diagnosis and by mortality status.

To examine the association of the various demographic and comorbid conditions with pressure ulcers, logistic regression was used. Each independent variable was first examined in a simple logistic regression model, and then a multivariable logistic regression model was determined using a backward model-building strategy. During this model building, the independent variable with the largest non-significant *p*-value was removed from the model. If the −2log(likelihood) test was non-significant and the AIC indicated a better fit, the independent variable remained out of the model, and the next independent variable with the highest non-significant *p*-value was examined for removal. Otherwise, the variable was retained in the model, and the independent variable with the second highest non-significant *p*-value was examined for removal. The final model contained all independent variables that were statistically significant or which improved the model fit. Odds ratios (OR) and 95% confidence intervals (CI) were determined.

Propensity scores for being diagnosed with at least one pressure ulcer were determined using all potential demographic and clinical comorbidities. Inverse propensity scores adjusted back to the sample size in each pressure ulcer group were then used as a weight in the Cox Proportional Hazards (CPH) modeling to account for characteristics such as the time of exposure to ESRD/dialysis.

The relationship between pressure ulcer diagnosis and survival was examined descriptively using Kaplan–Meier survival curves of those with and without pressure ulcers, and these curves were compared using a log-rank test to determine survival from the time of initiation of dialysis/diagnosis of ESRD.

To adjust for potential covariates and confounding, CPH modeling with adjusted inverse propensity score weights was used to examine the relationship between pressure ulcer diagnosis and mortality, controlling for the demographic and comorbid conditions discussed above. A pressure ulcer diagnosis and all potential covariates or confounders were first examined in simple CPH models, and then a similar backward model-building process was used to arrive at a final multivariable model. Hazard ratios (HR) and 95% CI were determined.

## 3. Results

### 3.1. Prevalence of Pressure Ulcer Diagnosis and Descriptive Parameters of the Cohort

For the 14-year study period, 291,871 out of 1,526,366 (19.1%) eligible patients were identified as having a pressure ulcer diagnosis (Table 1). Patients diagnosed with a pressure ulcer were more likely to be older, female, of white race, and non-Hispanic ethnicity and on hemodialysis with a graft or catheter access. They also exhibited a higher percentage of various clinical diagnoses, including spinal cord injury, malnutrition, myocardial infarction (MI), congestive heart failure (CHF), PVD, cerebral vascular disease (CVD), dementia, pulmonary disease, connective tissue disease, complicated and non-complicated diabetes, and paraplegia. Table 1 shows the descriptive statistics overall and by pressure ulcer status as well as the OR and 95% CI from the simple logistic regression models on pressure ulcers.

**Figure 1:**
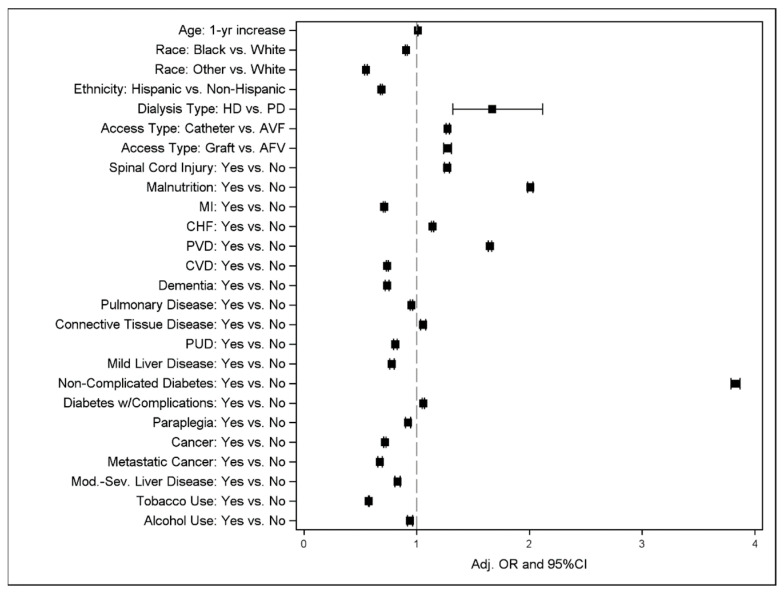
Forest plot of final multiple logistic regression model on pressure ulcers. Symbols show adjusted OR with 95% CI.

In the final model, demographic characteristics associated with a decreased risk of a pressure ulcer diagnosis included black race or other race (the reference is the white race) [adjusted OR = 0.91, 95% CI = 0.90–0.92 and OR = 0.55, CI = 0.54–0.56, respectively] and Hispanic ethnicity (OR = 0.69, CI = 0.68–0.70). In the multivariable analysis, patients with pressure ulcers were less likely to have a diagnosis of MI, CVD, dementia, pulmonary disease, cancer, metastatic cancer, paraplegia, peptic ulcer disease (PUD), mild or moderate to severe liver disease, and tobacco and alcohol use (Table 2, Figure 1).

Older age during the first ESRD service was associated with an increased risk of pressure ulcer diagnosis (OR = 1.01, CI = 1.01–1.01). Using hemodialysis versus peritoneal dialysis (OR = 1.67, CI = 1.32–2.12) was also associated with an increased risk of pressure ulcer diagnosis (Table 2). Clinical variables associated with an increased risk of pressure ulcer diagnosis included spinal injury (OR = 1.27, CI = 1.25–1.29) and malnutrition (OR = 2.01, CI = 1.98–2.03), as were CHF (OR = 1.14, CI = 1.13–1.15) and connective tissue disease (OR = 1.06, CI = 1.03–1.08). Diabetes with (OR = 1.06, CI = 1.05–1.07) or without (OR = 3.83, CI = 3.79–3.87) complications was also associated with an increased risk of pressure ulcers (Table 2). In patients with hemodialysis as their first dialysis modality, graft (OR = 1.27, CI = 1.24–1.31) and catheter (OR = 1.28, CI = 1.26–1.29) access types were associated with an increased risk of pressure ulcer diagnosis compared to arteriovenous (AV) fistula.

### 3.2. Mortality Analysis

**Figure 2:**
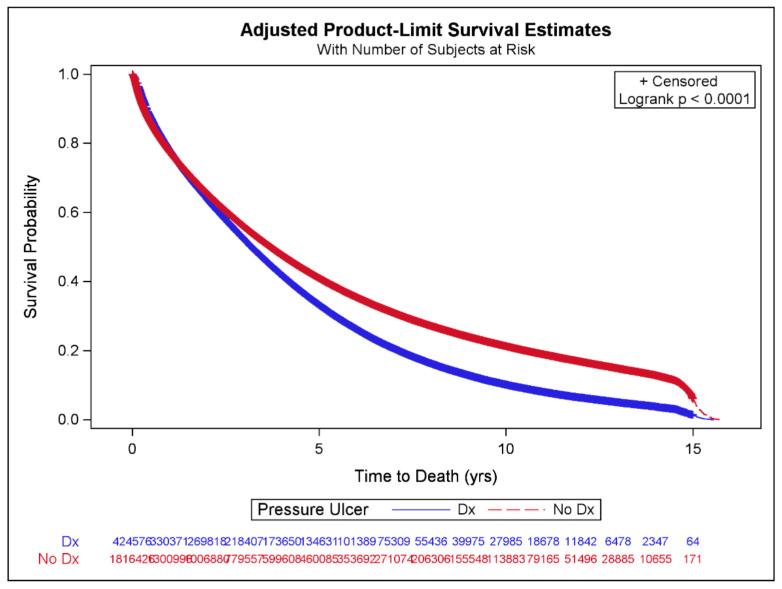
Kaplan–Meier survival curve by pressure ulcer status using adjusted inverse propensity score weights.

**Figure 3:**
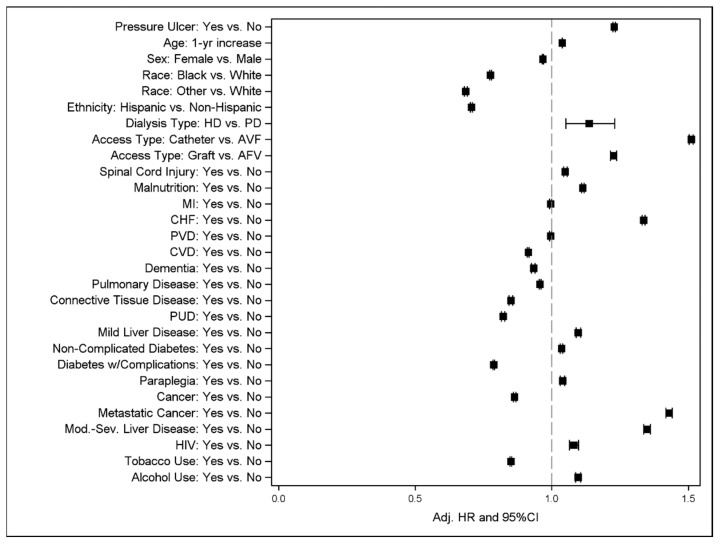
Forest plot of final multiple Cox Proportional Hazards model showing the effect of pressure ulcers and other variables on mortality when controlling for various covariates and confounders.

The Kaplan–Meier curves for patients with and without a pressure ulcer diagnosis are displayed in Figure 2. Survival was significantly worse for the group that had been diagnosed with a pressure ulcer compared to those who had not (log-rank test *p*-value < 0.001). After adjusting for potential confounders in multivariable CPH models, the presence of any pressure ulcer diagnosis was independently associated with increased mortality [adjusted hazard ratio (aHR) = 1.23, CI = 1.22–1.23] (Table 3, Figure 3). Female sex, black or other race, Hispanic ethnicity, and peritoneal dialysis were associated with a decreased risk of mortality, as were diagnoses of CVD, dementia, pulmonary disease, connective tissue disease, PUD, diabetes with complications, cancer, and tobacco use. Spinal cord injury and paraplegia, malnutrition, alcohol use, CHF, human immunodeficiency virus (HIV), mild and moderate to severe liver disease, and malnutrition were associated with an increased risk of mortality.

## 4. Discussion

### 4.1. Demographics

The aim of this study was to investigate the association between a diagnosis of pressure ulcers in ESRD patients and the risk of mortality. Using the USRDS database, we were able to include a large sample of US patients who initiated dialysis between 1 January 2005 and 31 December 2018. Our data indicate that of the 1,526,366 ESRD patients included in the study, 19.1% were diagnosed with pressure ulcers; for comparison, the global prevalence of pressure ulcers is estimated to be 14.8% [19]. When compared to patients without a pressure ulcer diagnosis, pressure ulcer patients tended to be female, on hemodialysis, and of white race. The association observed between increased age and the risk of pressure ulcers is supported by the literature indicating the delayed and impaired healing of wounds in the elderly [20]. Older populations are also more frail and have difficulty with mobility, which can further predispose them to the development of pressure ulcers [21]. In this study, pressure ulcers were observed more frequently in women than men, which is similar to prior studies on hospital and post-operative populations [22,23]. The current study also showed an increased risk of pressure ulcers in individuals of white race compared to those of black race. This finding is contrary to multiple studies in nursing home residents, which have all found an increased incidence of pressure ulcers in black compared to white individuals [24,25,26]. However, our results are consistent with a study suggesting that black patients have a greater number and a variety of comorbidities, including many of which we controlled in the current study, such as paralysis, CHF, and diabetes mellitus with complications [27,28]. Patients diagnosed with pressure ulcers were also less likely to be of other race. Because this other race category includes American Indians/Alaska Natives, Asians, Pacific Islanders/Native Hawaiians, Mid-East/Arabian, and the Indian sub-continent, this association is consistent with the results of a study in a nursing home population by Harms et al. in which a decreased prevalence of pressure ulcers in Asians and American Indians was found in comparison to all other races [29].

### 4.2. Comorbidities

In the present study, pressure ulcers were associated with multiple clinical risk factors, including spinal injury, malnutrition, cardiovascular disorders (CHF and PVD), connective tissue disease, liver disease, and diabetes with complications [diabetes mellitus with renal ophthalmic or neurological manifestations] and without complications. Some of these comorbidities, such as diabetes, PVD, and malnutrition, are commonly recognized to impair the process of wound healing [30]. Malnutrition is a known risk factor for pressure ulcers, which can contribute to poor wound healing via its effects on immune function and collagen synthesis [31]. Dialysis itself can also contribute to malnutrition in patients with ESRD, as the process may result in the loss of nutrients, including proteins; however, malnutrition in patients with ESRD can also be attributed to satiety, nausea, and vomiting, which is secondary to uremia, as well as increased resting energy expenditure secondary to inflammation [7,32,33,34].

Spinal cord injury is another known risk factor for pressure ulcers, as it leaves patients immobile, with an altered sense of pressure, and/or wheelchair-bound, increasing their opportunity to experience prolonged skin pressure, which promotes tissue ischemia [5]. Decreased mobility may even explain the increased incidence of pressure ulcers among the stroke population, as strokes can cause unilateral weakness, which can also impair movement and mobility [21]. In simple models, paraplegia and dementia were associated with an increased risk of pressure ulcer diagnosis, and upon controlling for demographic and clinical parameters, these disorders were no longer associated with pressure ulcer diagnoses.

Another known risk factor for pressure ulcers is cardiovascular disease, including MI, CHF, CVD, and PVD; whereas, in the present study, CHF and PVD were associated with a greater risk of pressure ulcer diagnosis, patients with pressure ulcer diagnosis were less likely to have an accompanying diagnosis of CVD and MI after controlling for demographics and multiple comorbidities, which could reflect a statistical artifact from other covariates in the analysis. CHF and PVD likely contribute to the development of pressure ulcers secondary to the decreased blood perfusion of tissues. In addition to decreased perfusion, a unique factor that might contribute to pressure ulcer development in patients with these diseases is edema. In patients with CHF (and PVD), edema can adversely affect the integrity of the skin and, thus, contribute to pressure ulcer incidence [21].

Interestingly, in the present study, pulmonary disease and tobacco use were less likely to be associated with a diagnosis of pressure ulcers. This result is contrary to the findings of a prior study in older ambulatory patients in whom chronic obstructive pulmonary disease was shown to be significantly associated with pressure ulcer development [35]. In a manner similar to cardiovascular disease, tissue hypoxia, secondary to inadequate oxygenation in patients with chronic obstructive pulmonary disease, could potentially explain the increased risk of pressure ulcers seen in these patients [21]. Likewise, cigarette smoking has also been associated with pressure ulcers via a similar mechanism of inadequate oxygenation of tissues due to smoking’s vasoconstrictive effect on the capillaries. In addition to inadequate oxygenation, smoking also has negative effects on wound healing [36]. Multiple studies in intensive care unit populations have found smoking to be significantly associated with pressure ulcers [36,37,38] in contrast to our findings. However, in a study examining pressure ulcers in veterans with a spinal cord injury, as well as another study in intensive care unit populations, no significant association was seen between smoking and pressure ulcer development [38,39], similar to the present study. As expected, an increased risk of pressure ulcers was observed in ESRD patients with diabetes with and without complications.

### 4.3. Mortality

Overall, this study indicates that the diagnosis of pressure ulcers in patients on dialysis is a significant independent risk factor for mortality in ESRD, leading to a reduced average survival time. This relationship between pressure ulcers and increased mortality remains even after controlling for various co-morbidities that are determined to be risk factors for mortality in the ESRD population, such as spinal cord injuries, malnutrition, CHF, liver disease, cancer metastasis, and diabetes.

As supported in the prior research of this population, black race, Hispanic ethnicity, and peritoneal dialysis (PD) were all protective against mortality. Despite having higher mortality in the overall US population, black race has historically been shown to be protective against mortality in patients with ESRD [40,41], as also seen in the present study. Although the causes of this apparent paradox are not entirely clear, it may be because those ESRD patients of black race start dialysis at a younger age and have a higher body mass index (BMI) [41]. A higher BMI has been associated with better survival in dialysis patients in several studies [42,43]. A recent study also suggested a possible correlation between improved survival in patients with apolipoprotein L1, a genetic variant common to African Americans and related to ESRD, when compared to patients with other causes of ESRD [44]. The protective association of Hispanic ethnicity on mortality despite their lower socioeconomic status is also somewhat unexpected but may be explained by the “salmon hypothesis”, which posits that ill foreign-born Hispanics may be more likely to return to their country of origin and, therefore, may not be represented in the data [45]. When comparing individuals on peritoneal dialysis versus those on hemodialysis in terms of mortality, it is important to note that patients who initiate PD rather than hemodialysis tend to have different demographics due to several factors, including physician selection bias. Thus, patients on the PD modality are typically younger, more educated, and have fewer comorbidities than patients on hemodialysis [46]. In fact, in a study by Wang et al., a similar survival was found between the modalities when inclusion was limited to only patients who were eligible for both hemodialysis and PD [47].

### 4.4. Limitations

The current study used administrative data from the USRDS, “a national data system that collects, analyzes, and distributes information about ESRD in the United States [48].” It is of utmost importance to recognize the limitations concerning the use of this dataset. First, retrospective studies inherently suffer from limitations associated with data quality, documentation accuracy, and missing or incomplete variables. These parameters can introduce bias and affect the reliability of the study’s findings. In our study, the presence of pressure ulcers was defined using ICD-9-CM and ICD-10-CM codes submitted from medical professionals to Medicare and not from clinical data; thus, certain clinical factors, such as BMI at the time of pressure ulcer diagnosis, might not be assessed. Information regarding the severity, stage, duration, and number of pressure ulcers was also not available and, therefore, not incorporated into the analysis, limiting the ability of this study to establish causality. In addition, our analysis did not account for the multiple diagnoses of pressure ulcers and/or a history of pressure ulcers nor for whether the pressure ulcer occurred in a hospital or outpatient setting. Second, we are unable to account for possible errors in diagnosis or missed diagnoses in the database. For example, calciphylaxis, a rare ischemic cutaneous wound that is nearly exclusive to ESRD patients, could conceivably be misdiagnosed as an unstageable pressure ulcer due to its necrotic appearance [49]. It is also important to note that the USRDS database does not provide data such as laboratory results or clinical observations, or a complete diagnostic history prior to dialysis; thus, there is the possibility that there are other confounding variables not controlled for in this population, particularly since USRDS patients might also have private insurance with claims that are not included in the USRDS database. Lastly, due to the retrospective nature of this study, we were unable to determine a cause-and-effect relationship between pressure ulcers and mortality in this ESRD population. Future prospective studies should be performed to better explore these relationships. In the present study, neither the cause of death nor the severity of pressure ulcers was considered, and future investigations should include these factors to better guide pressure ulcer management by healthcare practitioners. Despite these limitations, the large quantity of data compiled in the USRDS may lessen these concerns by providing considerable statistical power.

## 5. Conclusions

By controlling common comorbidities known to be risk factors for mortality, this study provides insight into the significance of pressure ulcers in ESRD and establishes pressure ulcers as a significant independent risk factor for mortality. To our knowledge, this study is the first to establish the risk factors for pressure ulcers specific to the ESRD population, which can hopefully better guide the management and implementation of preventative strategies for these patients. Indeed, these results underscore the importance of the early detection, prevention, and management of pressure ulcers in patients with ESRD. Healthcare providers should be vigilant in assessing and monitoring patients for the presence of pressure ulcers, particularly in those with advanced renal disease. The implementation of preventive strategies, such as regular repositioning, adequate nutrition, and appropriate wound care, could help mitigate the negative impact of pressure ulcers on patient outcomes. Our findings also suggest that continued studies, including prospective research on the topic of pressure ulcers in patients with ESRD, are warranted in order to further support this article’s current findings as well as to discover how best to prevent, diagnose, and treat pressure ulcers in this population. Nevertheless, the study serves as a reminder of the importance of preventive measures and timely intervention in managing pressure ulcers in patients with ESRD.

## Figures and Tables

**Table 1:** Descriptive statistics by pressure ulcer status and simple logistic regression results on pressure ulcers.

Variable	Level	Overall	Pressure Ulcer				
			Yes N = 291,871 (19.1%)	No N = 1,234,495 (80.9%)	OR	95% CI	*p*-Value
Age—mean (SD)		63.5 (14.9)	66.1 (13.5)	62.9 (15.1)	1.02	1.015–1.015	<0.0001
Sex—n (%)	Female	652,712 (42.8)	131,615 (45.1)	521,097 (42.2)	1.12	1.12–1.13	<0.0001
	Male	873,654 (57.2)	160,256 (54.9)	713,398 (57.8)			
Race—n (%)	Black	425,551 (27.9)	80,818 (27.7)	344,733 (27.9)	0.95	0.94–0.96	<0.0001
	Other	93,735 (6.1)	11,771 (4.0)	81,964 (6.6)	0.58	0.57–0.59	
	White	1,007,080 (66.0)	199,282 (68.3)	807,798 (65.4)			
Ethnicity—n (%)	Hispanic	230,176 (15.1)	36,488 (12.5)	193,688 (15.7)	0.77	0.76–0.78	<0.0001
	Non-Hispanic	1,296,190 (84.9)	255,383 (87.5)	1,040,807 (84.3)			
Dialysis Type—n (%)	HD	1,525,577 (99.9)	291,788 (99.9)	1,233,789 (99.9)	2.00	1.59–2.51	<0.0001
	PD	789 (0.1)	83 (0.1)	706 (0.1)			
Access Type—n (%)	Catheter	1,232,937 (80.8)	242,850 (83.2)	990,087 (80.2)	1.30	1.29–1.32	<0.0001
	Graft	49,772 (3.2)	10,418 (3.6)	39,354 (3.2)	1.41	1.37–1.44	
	AV Fistula	243,657 (16.0)	38,603 (13.2)	205,054 (16.6)			
Spinal Cord Injury—n (%)	Yes	118,314 (7.8)	30,617 (10.5)	87,697 (7.1)	1.53	1.51–1.55	<0.0001
	No	1,408,052 (92.3)	261,254 (89.5)	1,146,798 (92.9)			
Malnutrition—n (%)	Yes	201,231 (13.2)	65,145 (22.3)	136,086 (11.0)	2.32	2.30–2.34	<0.0001
	No	1,325,135 (86.8)	226,726 (77.7)	1,098,409 (89.0)			
MI—n (%)	Yes	343,272 (22.5)	69,624 (23.9)	273,648 (22.2)	1.10	1.09–1.11	<0.0001
	No	1,183,094 (77.5)	222,247 (76.2)	960,847 (77.8)			
CHF—n (%)	Yes	876,994 (57.5)	194,336 (66.6)	682,658 (55.3)	1.61	1.60–1.62	<0.0001
	No	649,372 (42.5)	97,535 (33.4)	551,837 (44.7)			
PVD—n (%)	Yes	483,472 (31.7)	130,363 (44.7)	353,109 (28.6)	2.02	2.00–2.03	<0.0001
	No	1,042,894 (68.3)	161,508 (55.3)	881,386 (71.4)			
CVD—n (%)	Yes	465,054 (30.5)	94,458 (32.4)	370,596 (300)	1.12	1.11–1.13	<0.0001
	No	1,061,312 (69.5)	197,413 (67.6)	863,899 (70.0.)			
Dementia—n (%)	Yes	60,040 (3.9)	12,633 (4.3)	47,407 (3.8)	1.13	1.11–1.16	<0.0001
	No	1,466,326 (96.1)	279,238 (95.7)	1,187,088 (96.2)			
Pulmonary Disease—n (%)	Yes	474,625 (31.1)	103,515 (35.5)	371,110 (30.1)	1.28	1.27–1.29	<0.0001
	No	1,051,741 (68.9)	188,356 (64.5)	863,385 (69.9)			
Connective Tissue Disease—n (%)	Yes	57,467 (3.8)	12,179 (4.2)	45,288 (3.7)	1.14	1.12–1.17	<0.0001
	No	1,468,899 (96.2)	279,692 (95.8)	1,189,207 (96.3)			
PUD—n (%)	Yes	90,918 (6.0)	17,438 (6.0)	73,480 (5.9)	1.00	0.99–1.02	0.6464
	No	1,435,448 (94.0)	274,433 (94.0)	1,161,015 (94.1)			
Mild Liver Disease—n (%)	Yes	79,556 (5.2)	13,465 (4.6)	66,091 (5.4)	0.86	0.84–0.87	<0.0001
	No	1,446,810 (94.8)	278,406 (95.4)	1,168,404 (94.7)			
Non-Complicated Diabetes—n (%)	Yes	670,686 (43.9)	204,373 (70.0)	466,313 (37.8)	3.85	3.81–3.88	<0.0001
	No	855,680 (56.1)	87,498 (30.0)	768,182 (62.2)			
Diabetes with Complications—n (%)	Yes	673,433 (44.1)	161,235 (55.2)	512,198 (41.5)	1.74	1.73–1.76	<0.0001
	No	852,933 (55.9)	130,636 (44.8)	722,297 (58.5)			
Paraplegia—n (%)	Yes	54,084 (3.5)	11,924 (4.1)	42,160 (3.4)	1.21	1.18–1.23	<0.0001
	No	1,472,282 (96.5)	279,947 (95.9)	1,192,335 (96.6)			
Cancer—n (%)	Yes	190,950 (12.5)	31,119 (10.7)	159,831 (12.9)	0.80	0.79–0.81	<0.0001
	No	1,335,416 (87.5)	260,752 (89.3)	1,074,664 (87.1)			
Metastatic Cancer—n (%)	Yes	55,018 (3.6)	7352 (2.5)	47,666 (3.9)	0.64	0.63–0.66	<0.0001
	No	1,471,348 (96.4)	284,519 (97.5)	1,186,829 (96.1)			
Moderate to Severe Liver Disease—n (%)	Yes	49,027 (3.2)	8166 (2.8)	40,861 (3.3)	0.84	0.82–0.86	<0.0001
	No	1,477,339 (96.8)	283,705 (97.2)	1,193,634 (96.7)			
HIV—n (%)	Yes	16,622 (1.1)	2786 (0.9)	13,836 (1.1)	0.85	0.82–0.89	<0.0001
	No	1,509,744 (98.9)	289,085 (99.1)	1,220,659 (98.9)			
Tobacco Use—n (%)	Yes	434,795 (28.5)	67,766 (23.2)	367,029 (29.7)	0.72	0.71–0.72	<0.0001
	No	1,091,571 (71.5)	224,105 (76.8)	867,466 (70.3)			
Alcohol Use—n (%)	Yes	61,348 (4.0)	9153 (3.1)	52,195 (4.2)	0.73	0.72–0.75	<0.0001
	No	1,465,018 (96.0)	282,718 (96.9)	1,182,300 (95.8)			

Abbreviations used: SD, standard deviation; HD, hemodialysis; PD, peritoneal dialysis; AV, arteriovenous; MI, myocardial infarction; CHF, congestive heart failure; PVD, peripheral vascular disease; CVD, cerebral vascular disease; PUD, peptic ulcer disease; HIV, human immunodeficiency virus.

**Table 2:** Full and final logistic regression models on pressure ulcers.

Variable	Level	Full			Final		
		OR	95% CI	*p*-Value	OR	95% CI	*p*-Value
Age	1-yr increase	1.01	1.01–1.01	<0.0001	1.01	1.01–1.01	<0.0001
Sex	Female vs. Male	1.00	1.00–1.01	0.3222			
Race	Black vs. White	0.91	0.90–0.91	<0.0001	0.91	0.90–0.92	<0.0001
	Other vs. White	0.55	0.54–0.56		0.55	0.54–0.56	
Ethnicity	Hispanic vs. Non-Hispanic	0.69	0.68–0.70	<0.0001	0.69	0.68–0.70	<0.0001
Dialysis Type	HD * vs. PD **	1.67	1.32–2.12	<0.0001	1.67	1.32–2.12	<0.0001
Access Type	Catheter vs. AVF	1.27	1.26–1.29	<0.0001	1.28	1.26–1.29	<0.0001
	Graft vs. AFV	1.27	1.24–1.31		1.27	1.24–1.31	
Spinal Cord Injury	Yes vs. No	1.27	1.25–1.29	<0.0001	1.27	1.25–1.29	<0.0001
Malnutrition	Yes vs. No	2.01	1.98–2.03	<0.0001	2.01	1.98–2.03	<0.0001
MI	Yes vs. No	0.71	0.71–0.72	<0.0001	0.71	0.71–0.72	<0.0001
CHF	Yes vs. No	1.14	1.13–1.15	<0.0001	1.14	1.13–1.15	<0.0001
PVD	Yes vs. No	1.65	1.63–1.67	<0.0001	1.65	1.63–1.67	<0.0001
CVD	Yes vs. No	0.74	0.73–0.75	<0.0001	0.74	0.73–0.75	<0.0001
Dementia	Yes vs. No	0.74	0.72–0.75	<0.0001	0.74	0.72–0.75	<0.0001
Pulmonary Disease	Yes vs. No	0.95	0.94–0.96	<0.0001	0.95	0.94–0.96	<0.0001
Connective Tissue Disease	Yes vs. No	1.06	1.03–1.08	<0.0001	1.06	1.03–1.08	<0.0001
PUD	Yes vs. No	0.81	0.80–0.83	<0.0001	0.81	0.80–0.83	<0.0001
Mild Liver Disease	Yes vs. No	0.78	0.76–0.80	<0.0001	0.78	0.76–0.80	<0.0001
Non-Complicated Diabetes	Yes vs. No	3.83	3.79–3.87	<0.0001	3.83	3.79–3.87	<0.0001
Diabetes with Complications	Yes vs. No	1.06	1.05–1.07	<0.0001	1.06	1.05–1.07	<0.0001
Paraplegia	Yes vs. No	0.92	0.90–0.95	<0.0001	0.92	0.90–0.95	<0.0001
Cancer	Yes vs. No	0.72	0.71–0.73	<0.0001	0.72	0.71–0.73	<0.0001
Metastatic Cancer	Yes vs. No	0.67	0.66–0.69	<0.0001	0.67	0.66–0.69	<0.0001
Moderate to Severe Liver Disease	Yes vs. No	0.83	0.81–0.85	<0.0001	0.83	0.81–0.85	<0.0001
HIV	Yes vs. No	1.02	0.98–1.07	0.3985			
Tobacco Use	Yes vs. No	0.58	0.57–0.58	<0.0001	0.58	0.57–0.58	<0.0001
Alcohol Use	Yes vs. No	0.94	0.92–0.97	<0.0001	0.94	0.92–0.97	<0.0001

* HD = hemodialysis, ** PD = peritoneal dialysis.

**Table 3:** Descriptive statistics by mortality with simple and final CPH models on mortality, weighted using adjusted inverse propensity score weights.

Variable	Level	Descriptive Statistics		Simple CPH Models			Final CPH Model		
		Died	Alive	HR	95% CI	*p*-Value	HR	95% CI	*p*-Value
Time to Death/Period of Observation-mean (SD)		2.9 (3.4)	4.9 (4.6)						
Pressure Ulcer—(%)	Yes	25.2	6.4	1.28	1.27–1.28	<0.0001	1.23	1.22–1.23	<0.0001
	No	74.8	93.6						
Age—mean (SD)		67.3 (16.4)	56.8 (17.9)	1.04	1.041–1.041	<0.0001	1.04	1.04–1.04	<0.0001
Sex—(%)	Female	43.5	41.4	1.04	1.03–1.04	<0.0001	0.97	0.96–0.97	<0.0001
	Male	56.5	58.6						
Race—(%)	Black	25.4	32.5	0.70	0.69–0.70	<0.0001	0.78	0.77–0.78	<0.0001
	Other	5.2	7.8	0.67	0.66–0.67		0.69	0.68–0.69	
	White	69.4	59.7						
Ethnicity—(%)	Hispanic	12.4	19.4	0.69	0.68–0.70	<0.0001	0.71	0.70–0.71	<0.0001
	Non-Hispanic	87.7	80.6						
Dialysis Type—(%)	HD *	99.9	99.9	1.36	1.26–1.47	<0.0001	1.14	1.05–1.23	0.0014
	PD **	0.1	0.1						
Access Type—(%)	Catheter	82.5	77.2	1.44	1.43–1.44	<0.0001	1.51	1.50–1.52	<0.0001
	Graft	3.3	3.2	1.26	1.25–1.27		1.23	1.21–1.24	
	AV Fistula	14.1	19.6						
Spinal Cord Injury—(%)	Yes	10.4	3.7	1.09	1.08–1.10	<0.0001	1.05	1.04–1.06	<0.0001
	No	89.6	96.3						
Malnutrition—(%)	Yes	18.4	4.9	1.26	1.26–1.27	<0.0001	1.11	1.11–1.12	<0.0001
	No	81.6	95.1						
MI—(%)	Yes	28.4	12.7	1.14	1.14–1.15	<0.0001	1.00	0.99–1.00	0.0551
	No	71.6	87.3						
CHF—(%)	Yes	66.9	41.0	1.51	1.50–1.51	<0.0001	1.34	1.33–1.34	<0.0001
	No	33.1	59.0						
PVD—(%)	Yes	37.1	22.1	1.14	1.13–1.14	<0.0001	1.00	0.99–0.99	0.0293
	No	62.9	77.9						
CVD—(%)	Yes	35.7	22.1	1.06	1.06–1.07	<0.0001	0.91	0.91–0.92	<0.0001
	No	64.3	77.9						
Dementia—(%)	Yes	5.5	1.3	1.27	1.26–1.28	<0.0001	0.93	0.93–0.94	<0.0001
	No	94.5	98.7						
Pulmonary Disease—(%)	Yes	38.1	20.2	1.11	1.11–1.12	<0.0001	0.96	0.95–0.96	<0.0001
	No	61.9	79.8						
Connective Tissue Disease—(%)	Yes	4.2	3.4	0.81	0.80–0.82	<0.0001	0.85	0.84–0.86	<.0001
	No	95.9	96.6						
PUD—(%)	Yes	7.1	4.2	0.86	0.86–0.87	<0.0001	0.82	0.82–0.83	<0.0001
	No	92.9	95.8						
Mild Liver Disease—(%)	Yes	6.5	3.3	1.09	1.08–1.09	<0.0001	1.10	1.09–1.11	<0.0001
	No	93.5	96.7						
Non-Complicated Diabetes—(%)	Yes	50.7	31.5	1.01	1.01–1.01	<0.0001	1.04	1.03–1.04	<0.0001
	No	49.3	68.6						
Diabetes with Complications—(%)	Yes	46.0	42.0	0.81	0.81–0.81	<0.0001	0.79	0.78–0.79	<0.0001
	No	54.0	58.0						
Paraplegia—(%)	Yes	4.5	2.1	1.01	1.01–1.02	0.0009	1.04	1.03–1.05	<0.0001
	No	95.6	97.9						
Cancer—(%)	Yes	15.6	7.7	1.14	1.14–1.15	<0.0001	0.86	0.86–0.87	<0.0001
	No	84.4	92.3						
Metastatic Cancer—(%)	Yes	5.2	1.0	1.49	1.48–1.50	<0.0001	1.43	1.42–1.44	<0.0001
	No	94.8	99.0						
Moderate to Severe Liver Disease—(%)	Yes	4.3	1.5	1.25	1.24–1.26	<0.0001	1.35	1.34–1.36	<0.0001
	No	95.7	98.5						
HIV—(%)	Yes	1.1	1.3	0.70	0.69–0.71	<0.0001	1.08	1.06–1.10	<0.0001
	No	98.9	98.8						
Tobacco Use—(%)	Yes	31.4	24.8	0.86	0.86–0.87	<0.0001	0.85	0.85–0.85	<0.0001
	No	68.6	75.2						
Alcohol Use—(%)	Yes	4.5	3.4	0.92	0.92–0.93	<0.0001	1.10	1.09–1.11	<0.0001
	No	95.5	96.6						

* HD = hemodialysis, ** PD = peritoneal dialysis.

## Data Availability

The data underlying this article were provided by the United States Renal Data System (USRDS) under a data use agreement. Data will be shared on request to the corresponding author with the permission of the United States Renal Data System. The contents of this article do not represent the views of the Department of Veterans Affairs or the United States Government. The data reported here have been supplied by the USRDS. The interpretation and reporting of these data are the responsibility of the authors and in no way should be seen as official policy or an interpretation of the United States Government.

## Supplementary Materials

The following supporting information can be downloaded at: https://www.mdpi.com/article/10.3390/life13081713/s1, Table S1: ICD-9-CM and ICD-10-CM codes used for the diagnosis of Pressure Ulcer; Table S2: ICD-9-CM and ICD-10-CM diagnosis codes for controlled comorbidities included in the Charlson Comorbidity Index; Table S3: ICD-9-CM and ICD-10-CM diagnosis codes for controlled comorbidities not included in the Charlson Comorbidity Index.
