# Supplementary material for: Pressure Ulcer Diagnosis Is Associated with Increased Mortality in Patients with End-Stage Renal Disease: A Retrospective Study
Source: Life (Basel). 2023 Aug 9;13(8):1713. doi: 10.3390/life13081713 (PMC10456114; doi:10.3390/life13081713)
Supplement: Supplementary file 1 [file life-13-01713-s001.zip › life-2494375-supplementary.pdf]

Supplementary Table S1. ICD-9-CM and ICD-10-CM codes used for the diagnosis of Pressure Ulcer		
**AAA Diagnosis**	**ICD-9-CM**	**ICD-10-CM**
Pressure Ulcer	707	L89

Supplementary Table S2. ICD-9-CM and ICD-10-CM diagnosis codes for controlled comorbidities included in the Charlson Comorbidity Index		
**Variable**	**ICD-9-CM Codes**	**ICD-10-CM Codes**
Acute MI	410, 412	I21, I22, I252
Congestive Heart Failure	428	I50
Peripheral Vascular Disease	441, 4439, 7854, V434	I71, I739, I790, R02, Z958, Z959
Cerebral Vascular Disease	430–438	G46, G450, G451, G452, G454, G458, G459, I60, I61, I62, I63, I64, I65, I66, I670, I671, I672, I674, I675, I676, I677, I678, I679, I681, I682, I688, I69
Dementia	290	F00, F01, F02, F051
Pulmonary Disease	490, 491, 492, 493, 494, 495, 496, 500, 501, 502, 503, 504, 505	J40, J41, J42, J43, J44, J45, J46, J47, J60, J61, J62, J63, J64, J65, J66, J67
Connective Tissue Disorder or Rheumatologic Disease	7100, 7101, 7104, 7140, 7141, 7142, 71481(now 5171), 725	M050, M051, M052, M053, M058, M059, M060, M063, M069, M32, M332, M34, M353
Peptic Ulcer	531, 532, 533, 534	K25, K26, K27, K28
Liver Disease	5712, 5714, 5715, 5716	K702, K703, K717, K73, K740, K742, K743, K744 K745, K746
Diabetes	2500, 2501, 2502, 2503, 2507	E101, E105, E109, E111, E115, E119, E131, E135, E139, E141, E145, E149,
Diabetes with Complications	2504, 2505, 2506	E102, E103, E104, E112, E113, E114, E132, E133, E134, E142, E143, E144
Paraplegia	342, 3441	G041, G81, G820, G821, G822

**Supplementary Table S2. ICD-9-CM and ICD-10-CM diagnosis codes for controlled comorbidities included in the Charlson Comorbidity Index**		
**Variable**	**ICD-9-CM Codes**	**ICD-10-CM Codes**
Renal Disease	582, 5830, 5831, 5832, 5833, 5834 5835, 5836, 5837, 585, 586, 588	N01, N03, N052, N053, N054, N055, N056, N072, N073, N074, N18, N19, N25
Cancer	140-172, 174-195, 200, 201, 202, 203, 204, 205, 206, 207, 208	C0, C1, C2, C3, C40, C41, C43, C45, C46, C47, C48, C49, C5, C6, C70, C71, C72, C73, C74, C75, C76, C80, C81, C82, C83, C84, C85, C883, C887, C889, C900, C901, C91, C92, C93, C940, C941, C942, C943, C9451, C947, C95, C96
Metastatic Cancer	196, 197, 198, 1990, 1991	C77, C78, C79, C80
Severe Liver Disease	5722, 5723, 5724, 5728	K721, K729, K766, K767
HIV/Aids	042, 043, 044	B20, B21, B22, B23, B24

**Supplementary Table S3. ICD-9-CM and ICD-10-CM diagnosis codes for controlled comorbidities not included in the Charlson Comorbidity Index**		
**Variable**	**ICD-9-CM Codes**	**ICD-10-CM Codes**
Spinal cord injury	952	S14, S24, S34
Malnutrition	260, 261, 262, 263, 263.1, 263.2, 263.8, 263.9, 799.4	E40, E41, E42, E43, E44.0, E44.1, E45, E46, R64
Smoking	305.1, V15.82	F17.200, Z87.891
Alcohol Use	305, 305.01, 305.02, 305.03, 303.9, 303.92, 303.93	F10.10, F10.11, F10.20, F10.21
